# Remarks on Fracture of the Cranium

**Published:** 1852-03

**Authors:** John Hardin

**Affiliations:** Louisville, Ky.


ART. I.—*Remarks on Fracture of the Cranium.* By JOHN HARDIN, M. D., of Louisville, Ky.

*Case* 1.—In the spring of 1844, Tom, a negro man between thirty and forty years of age, received a blow from the back of a hoe in the hands of a fellow servant, felling him to the ground. The medical gentleman who saw him on the same day, found the scalp laid open to the bone, and, as he supposed, a piece of the outer plate of the cranium chipped off. As there were no symptoms of cerebral compression, the wound was simply dressed and soon healed. In a few months, however, the patient became the subject of epilepsy and insanity. In October of the same year, Dr. D. P. White, of Greensburg, into whose hands the case had passed, requested my advice and assistance. After an examination we concurred in the propriety of using the trephine, which was done in the following manner: A crucial incision was made, and the flaps turned back, exposing a depression large enough to admit the end of the thumb and about half an inch in depth; which was filled with a substance of recent growth, of a fibrous structure. The crown of the largest trephine barely included the whole of the depressed portion. Upon sawing through and removing this, there were found numerous spicula pressing upon the dura mater, none of them, however, so far as could be ascertained, penetrating that membrane. These were all carefully removed. The dura mater was of a deeper red color than natural, evincing circumscribed, chronic inflammation. The recovery of the patient from epilepsy and insanity was complete, in a few weeks, but his general health, which had become very much impaired, was not restored, and a year afterwards he became the subject of paralysis of the lower extremities with dropsical effusions, and he died about two years after receiving the injury. As he lived at a distance from town, and his case was in a great measure lost sight of, no autopsy was made.

*Case* 2.—Nov. 30th, 1844, a lad, aged sixteen years, the son of William O. Johnston, had fallen, five months previously, from an apple tree and received, in the superior internal portion of the orbit, a hard seasoned sprout which was growing at the root of the tree. It was supposed that as he arose the snag was withdrawn, leaving a small wound through the eye-lid. This wound remained open and continued to discharge matter for some weeks, when it was ascertained that the foreign body was still lodged in the cranium. It was promptly removed and the wound healed; but soon afterwards, the young man was seized with epileptic convulsions, and subsequently passed into a furious insanity. At this stage of his malady, Dr. Hodgen, of Campbellsville, was called to attend him, and he requested my advice in the case. Not being apprised of the extent of the injury, bnt seeing that the brain was greatly diseased, we determined on an exploring operation. At this period the patient had from fifteen to twenty convulsions daily. An inverted Λ incision was made on the forehead above the injury, the flap turned down so as to expose the superciliary ridge, and upon the verge of this the centerpin was fixed, so as to remove a little more than half a circle of bone; when this was removed, the superior orbitar plate was found to be in a state of comminuted fracture; the fragments were carefully extracted; the internal wall of the frontal sinus being in a state of integrity was not interfered with; the membrane lining the sinus was in a state of subacute inflammation, and the operation was consequently very painful. The flap being replaced, water dressings were applied; the young man had but one fit after the operation, and four years afterwards when I last heard from him, he was in the enjoyment of sound health of body and of mind.

*Case* 3.—Oct. 31st, 1845, I was called to see the daughter of Mr. Romeyn, aged about ten or twelve years, who had just received a kick from a horse, producing a compound fracture of the anterior, inferior portion of the right parietal bone; this fracture was curvilinear and two and a half inches in length, the lower portion underlying the upper, and the fracture of the internal plate being considerably larger than that of the external. There were no constitutional symptoms indicating compression of the brain; no stertor, nor insensibility; but the depression was very great, and instructed by the disastrous effects following depressed bone without constitutional symptoms in previous cases, I could not, with a good conscience, leave my patient to the chances of a similar termination. I considered it as nearly certain, that if the constitutional evidences did not supervene within a few days, epilepsy would be the ultimate result; and under these impressions of the case, I resolved to elevate the depressed bone, and proceeded to place the centerpin of the trephine on the firm edge of the fracture, and removed a little more than a half circle, so that I might be able to get the elevator under the edge of the depressed portion; but after having accomplished this, I still could not raise the bone until, with Hey's saw, I removed the margin along the whole length of the fracure. After this was done, no further difficulty was experienced. Water dressings were applied, and the antiphlogistic regimen instituted, and the case progressed without an untoward symptom; the young lady has ever since enjoyed uninterrupted health.

*Case* 4—On the 11th day of November, 1846, I was called to see a colored boy fifteen years old, a striker in a blacksmith's shop, the property of Micajah Hilliard. He had sustained a compound fracture of the parietal bone, midway between its centre and the posterior, superior angle. This injury was from the hammer-like chisel with which smiths make the groove in horse shoes, which, from an awkward stroke of his own, flew off the helve and struck him on the head. A piece of bone the average diameter of which was about half or three quarters of an inch, of an irregular circular shape, was entirely detached and forced down upon the brain; and, as is usual, the fracture of the internal was greater than that of the external plate, owing to which circumstance it could not be removed except by the trephine. There were no constitutional symptoms present, but apprehending results such as attended the first two cases reported, and encouraged by the happy termination of the second and third, without hesitation, I applied the instrument, and removed enough bone to enable me to extract the fractured portion. Water dressings were applied, and antiphlogistic treatment instituted, and the patient recovered without a bad symptom. Last spring I saw him in perfect health, four years and a half having elapsed since he received the injury.

*Case* 5.—A negro boy, aged twelve or thirteen years, the property of Mr. Perrick, was thrown from a horse on the 12th of February, 1847, and when found two or three hours afterwards, was insensible. The persons who found him, supposed that he had been thrown against a large tree, near which he was lying. Dr. T. Q. Walker saw him on the same day, and on the 13th, requested my assistance, as he had determined to trephine; there was fracture of the parietal bone, with depression, profound coma with feeble circulation; a state of case which had existed from the time he was discovered. So profound was the insensibility, that he gave very slight indications of pain under the operation; the fractured bone was removed; no laceration of the dura mater was observed, nor was there any extravasation of blood above or beneath it that could be discovered. The operation appeared to be unproductive of any result, either good or evil; no change could be perceived in the condition of the patient, and on the 14th he died, without a moment's consciousness from the time of receiving the injury until his death. The case of this boy like the first, occurred at a distance from town, and he was buried before I heard of his death, so that no autopsy was made. There was probably injury, with extravasation deep within the brain, perhaps at its base. The spinal column or thoracic viscera may have suffered irreparable injury. The boy was so inanimate that nothing could be discovered beyond the visible injury, and such profound coma as might easily result from it. This case is not reported because of any thing intrinsically interesting in it, nor because it is illustrative of the views which I shall advance in relation to injuries of this nature, but because I think it right to publish unsuccessful as well as successful cases; and it may become useful at some future time, in the making up of statistics.

*Case* 6.—On the 18th of the same month (Feb'y, 1847), I was requested to see Daniel Akin, who the night previously had received a blow on the forehead with the back part of an axe, causing a fracture running obliquely from a point a little external to the centre of the superciliary ridge of the right side, upwards and inwards very nearly to the median line of the os frontis, and extending upwards about two-thirds the height of the forehead. The upper end of the fractured portion was deeply depressed, the skin over it was unbroken, and there were no constitutional symptoms of compressed brain; but although these were absent, the fact that there was actual compression was too palpably evident to be doubted for a moment. Bearing in mind the unhappy results of leaving a piece of depressed bone on the brain in some of the foregoing instances, and encouraged by the success in some others, I determined to elevate the bone, notwithstanding that I should thereby convert a simple into a compound fracture. Dr. Eastland, who was present with me, concurred in the propriety of the course. An inverted Λ incision was made and the flap turned down over the eye, so as to expose the seat of injury. In order to get the elevator under the edge of the depressed bone, it was necessary to remove a part out of the upper edge of the fracture, for which purpose the centrepin was fixed upon its margin, and, as in two of the other cases, a little more than half a circle removed. The elevator was now introduced, and the depressed portion raised, though not without the exercise of considerable force. The integrity of the dura mater was not disturbed. Water dressings and the antiphlogistic regimen were instituted, and Mr. Akin recovered without any bad symptoms, and has remained well up to this time.

It will be observed that the treatment instituted in some of the foregoing cases is in direct opposition to that impressively inculcated by nearly all writers on surgery. They teach, that the trephine should not be used in any case of fractured skull, no matter whether the fracture be compound or simple, and no matter how great the depression of bone, unless there be constitutional symptoms of compressed brain. Thus, Mr. Samuel Cooper says, "Even when the fracture is depressed, it is not necessary [to trephine] unless there are evident signs that the degree of pressure thus produced on the brain is the cause of existing bad symptoms." Mr. Abernethy's remarks are to the same point; he says, "Whenever the patient retains his senses perfectly, I should think it improper to trephine him, unless symptoms arose that indicated the necessity of it." Mr. Cooper says again, "If then the violence of the symptoms is not always in proportion to the compression, but is sometimes considerable when the pressure is slight, every surgeon cannot be too fully impressed with the following truth, that *existing symptoms of dangerous pressure on the brain, can alone form a true reason for perforating the cranium.*" Sir Astley Cooper says, "In simple fracture, when it is attended with symptoms of injury of the brain, deplete before you trephine; and when it is unattended with such symptoms, deplete merely, and do not divide the scalp. If the fracture be compound, the treatment must be very different; because a compound fracture is very generally followed by inflammation of the brain; and it will be of little use to trephine when inflammation is once produced. If the inflammation come on, the patient will generally die, whether you trephine or not."

"The rule (he continues) which I always follow is this: When I am called to a compound fracture with depres sion, which is exposed to view, whether symptoms of injured brain exist or not, I generally use an elevator, and very rarely the trephine. I put the elevator under the bone, raise it, and if it has been comminuted, remove the small pieces of bone." This practice is doubtless the most proper in cases where it is practicable, but the fracture in the inner table is, so generally, larger than that of the external, that it is impossible to introduce an elevator beneath the fractured portion, without first removing a part either with the trephine or Hey's saw. It seems to me that writers too commonly state the impropriety of trephining in simple fracture without depression, and then make a sweeping application of the principle to fractures with depression. The dangers of inflammation resulting from compound fractures with depression enumerated by Sir Astley, exist also, though, in a less degree, in simple fractures with depression. Indeed it appears to me, that the hazards of the operation itself are greatly exaggerated by authors, while the dangers of leaving a sharp-edged bone pressing upon the brain and irritating the dura mater, are mitigated. Mr. Samuel Cooper says, "Of the propriety of using the elevator in such cases [those of compound fracture with depression] and also of taking away loose fragments, there cannot be a doubt; but many surgeons object (and I confess myself one of the number) to saw out a portion of the skull while the patient is free from urgent symptoms. I believe, also, that the inflammation, when it does arise, is mostly the effect of the violence itself, not of the depression of the bone, and, therefore, more likely to be increased than prevented by the application of the trephine. I think a better reason for elevating the bone, when it is exposed, and there are no bad symptoms, is the fact, that many patients, after their recovery from the imminent danger of the accident, become subject, whenever the circulation is hurried, to insanity, epilepsy, &c. Yet, here it is to be considered, that it may be quite time enough to trephine, when such ills follow the continuance of the depression, and that, perhaps, the operation would then be in itself less dangerous, inasmuch as the tendency to inflammation, arising from the first violence must now have subsided."

If, as suggested in the foregoing extract, the greatest danger of inflammation is from the violence and not from the depression, it seems to me an additional reason for removing the depressed bone; that is, if the danger of inflammation from the violence is great, that danger must be greatly increased by the continued presence and pressure of the depressed bone—confessedly, an active, exciting cause of inflammation. And the doctrine advanced in the latter part of the extract, that even in cases where insanity, epilepsy, &c. follow, it may be better to wait, as the operation may be considered less dangerous in itself, when by lapse of time the danger from the first violence has passed away, is to my mind worse than questionable. I regard it as highly injudicious. In the first place, if the operation should be performed, at the time of the injury, inflammation will not have commenced, and can nearly always be prevented from running to a dangerous height, or indeed to any degree, beyond that necessary to the reparative process; whereas, if we wait until "insanity, epilepsy, &c." supervene, we are sure to find the meninges, at least, in a state of subacute inflammation. Then, as an additional objection to waiting for the coming on of insanity, epilepsy, &c., what assurance have we, that after the removal of the original cause, the malady may not be continued by the disease already engendered in the brain and its membranes, or by some obscure cause which has sometimes been termed "habit?" For my own part, I would greatly prefer to run the risk of dying of the proper and rational means of cure, than of becoming the subject of insanity, epilepsy, &c. In every case of pressure on the brain, there is hazard of inflammation, and where the cause of that pressure can be recognized, it appears to be the plainest dictate of common sense to remove it, and not leave that remedy, which is the only sure one, and should be first, to be used as a dernier resort. It is a matter of notoriety, that in cranial fractures the depression is greater than external appearances would indicate, from the fact, that the fracture is greater in the internal than external plate, and that surgeon would deceive himself and fail to discharge his duty to his patient, who should come to the conclusion, that notwithstanding there was a visible depression of the outer table, yet there might not be anything very serious within. I know that Sir Astley Cooper has said that, frequently, the outer table may exhibit considerable depression, when it is only depressed into the diploë, while the inner table remains uninjured; but unless the diploë was thicker in those days than it is at present, I hope I shall be excused if I should be a little sceptical as to the fact.

Sir Benj. Brodie lays down this general rule: "That if the depression be exposed in consequence of a wound in the scalp, let the surgeon apply the trephine, and elevate the depression; but if there be a depression without a wound of the scalp, in consequence of the accident, let him not make such a wound by an operation."

Dr. Gibson teaches that, "It seldom happens, that a mere depression of the bone, unattended by other injury, will give rise to severe symptoms, or to such as characterize a compression of the brain." And, again, after enumerating the symptoms of compression, and recommending the employment of bleeding, purging, and if these fail, then the trephine, he says: "The same observation will apply to fracture with depression, always recollecting, however, that the *symptoms* call for the operation, and not the mere fracture; and that the skull, if the brain be uninjured, will often unite like any other fractured bone, even although the depression be left. On the other hand, it should be stated, that a depressed fracture, which, at first, did not interfere with the functions of the brain, will sometimes give rise to inflammation of the dura mater or brain, and subsequently to compression, from the formation of matter; *and that these consequences, might have been prevented, perhaps, by a timely operation.* But a great deal of judgment will be required, to enable the surgeon to anticipate such consequences." Verily, I think, that the surgeon's judgment must be greatly biased by hope, who would not anticipate them, as the ordinary and legitimate consequences of such unremedied injuries. In another place, the same distinguished surgeon says: "With the exception of the occipital bone, the trephine may be applied with equal effect, and, comparatively, with little danger, to any part of the skull." A bone depressed upon the brain, even although there be no immediate, constitutional symptoms of compression, is confessedly a condition of great danger---danger of exciting, in a brief space of time, inflammation ending in suppuration, compression and death, or falling short of this—danger of producing chronic inflammation, or at least, morbid sensibility, terminating in insanity, epilepsy, &c., while the operation necessary to raise the depressed bone, is one, confessedly, of comparatively little danger; and yet we are taught by "line upon line and precept upon precept," that we should forego the lesser and encounter the greater danger.

But hear another authority. Miller says: "The question arises, whether the continued pressure of a smooth portion of depressed bone, or the injury and violence sustained by the parts, in the performance of the elevating operation, is the more likely to excite an untoward amount of vascular action? Experience has answered to the effect, that the greater risk is encountered by recourse to operation, and, consequently, the rule is, to refrain from operation in all cases of ordinary depressed fracture, in which symptoms of compression do not exist." It occurs to my mind, that this extract contains one or two fallacies, which it may be material to understand, in coming to a right conclusion on this question. The first statement which strikes me as fallacious, is couched in the word "*smooth* portion of depressed bone." Now, all the depressed portions of bone that I have seen, although they may have a smooth surface, have yet had sharp, irregular edges, against which the cranial contents, solids and fluids, constituting as they do, a plenum, would necessarily press. The other statement, which I regard as incorrect is that "experience has answered to the effect, that the greater risk is encountered by recourse to operation." Whose experience has so answered it? After diligent search, I can find no statistics to any such effect. True it is, that if we regard the statistics of the operations performed in hospitals, where erysipelas is so apt to prevail, and where, from the teachings of Abernethy, Cooper, Brodie and others, the operation is postponed until mischief, often irreparable, has been produced, we shall find them against the operation; but if we take those cases that have been operated on, soon after the injury has been sustained, and before the supervention of inflammation, coma, convulsions, &c., I apprehend that their results would be in our favor. Indeed, Sir Astly Cooper, Sir B. Brodie and a few other surgeons were brought to recommend the removal of depressed bone, where there was a wound in the integuments, but were still opposed to this procedure if there was no wound in the scalp! Is it not passing strange that an incision should have been regarded as of so much importance as to change the rational and radical method of cure? The operation itself is not one of a very formidable and dangerous character; in its performance, no violence need be done to the brain, and very little to its investing membranes; nor can it be believed, that the danger of a brief exposure of the wound to the atmosphere is at all comparable to that of leaving a bone depressed. Dr. Pancoast, after noticing the directions to trephine immediately, if the fracture and depression are attended with an external wound, says: "So little a matter, however, is the existence or non-existence of an external wound in so serious an affection, that the admission would seem to imply that a more frequent use of the trephine would be found advantageous; and Velpeau, in a more decided tone, advocates its employment."

Prof. Dudley, in his admirable paper on injuries of the head, published in the first volume of the Transylvania Journal of Medicine, says: "The experience which time and circumstances have afforded in injuries done the head, induces me to depart from the commonly received principles by which surgeons are governed in regard to the use of the trephine. In skilful hands the operation, beyond the atmosphere of large cities, is neither dangerous in its consequences, nor difficult in the execution." And, again, after an interesting train of most judicious remarks on the effects of pressure on various parts of the body, he further says: "Since the subordinate parts of the system are familiarly known to make severe complaint, when kept under the influence of undue pressure, in place of feeling embarrassment at the task of showing the necessity at all times of relieving the brain from a similar condition, whether morbid manifestations be present or otherwise, it rather appears to constitute matter of astonishment, that arguments in favor of removing pressure from any portion of its surface, should be thought necessary, in this enlightened period of the profession. That great violence is sometimes done without sensibly impairing any of the faculties, is admitted. It is matter of equal notoriety, however, that cases frequently occur, where injuries, apparently the lightest in character, are followed by the most disastrous consequences." And, yet, notwithstanding the clearness and force of these arguments, Professor Dudley says, in laying down rules, governing a decision, for or against the operation: "A fracture accompanied by a depression of a portion of the skull, wherein the integuments are wounded, while a union by the first intention can be effected, and in which the faculties, corporeal and intellectual, remain unimpaired, presents a case, which according to sound principles in surgery, forbids an operation with the trephine." This essay of Dr. Dudley is one of the most interesting that I have ever met with on this subject, and notwithstanding the rule for the operation just quoted, the reasoning throughout the whole paper shows clearly the propriety of removing pressure, in all cases, where it may exist. The old doctrine, that the trephine should be used in every case of fracture, even where there is no depression, but a mere fissure, has been very properly repudiated; but in avoiding Scylla, on the one side, may we not have run upon Charybdis, on the other? It is reported of an eminent physician that he used to say in his lectures, that the difference between himself and the professor of surgery, in the treatment of urinary calculus, was this, that the latter cured the affection by removing the stone, while he accomplished it by attention to the secretions of the liver, and left the stone in the bladder. About the same difference exists between those cases of depressed cranium, cured by elevating the depression, and those cured (?) without such elevation.

I know of no author who is so explicit in his advocacy of the trephine, both as a curative and prophylactic agent as Chelius; and as his work was not translated and published in English until recently, and is not now to be found in many libraries, I hope I shall be excused for extracting one of his rules. He says: "Fractures with depression of the skull always require immediate trephining, although unaccompanied with symptoms of pressure and irritation of the brain. These occur earlier or later, and then no benefit is derived from the operation. The object of trephining is to raise the depressed bone, to remove the extravasation and splinters of bone, and to prevent after-collections in the cavity of the skull. Trepanning is only superfluous when, from the condition of the wound, it is possible to remove carefully the splinters or completely broken off pieces, and to let the extravasation escape. It may be hoped in children who have fracture with impression, that with proper antiphlogistic curative means, nature will accommodate the brain to the pressure, if the indent be not upon a blood sinus, in which case it must be at once trepanned."

Upon this important question the profession stands thus: One party, (and this including the majority of English and American surgeons) restrict the use of the trephine to those cases in which constitutional symptoms have arisen, and justify themselves in declining to operate sooner by the undeniable fact, that some cases get well and remain well without the operation, and that therefore it is right, in each individual case, to wait and see if this will not be the result; and in order to secure it, use vigorously the antiphlogistic treatment. While the other party, including Petit, Pott, Chelius, and, with slight restrictions, Dudley and others, contend that in all cases of fracture with depression, we ought to apprehend and therefore guard against the worst that can happen; and that inasmuch as the operation is not in itself very highly dangerous, but that the secondary symptoms resulting from the accident are imminently perilous; and that as the continued pressure of the depressed bone is a sufficient cause to produce these secondary affections, therefore it is right to free the brain from such pressure; if it can be done with the elevator it is well; if it cannot, then the trephine must be used. They suppose that in most cases, after the supervention of inflammation, trephining would fail to cure; and that although some cases do well without the operation, the cure may be more confidently expected, and the patient better protected from ulterior bad results when it has been obtained by the removal of the offending bone.

It would not be philosophical to deduce a general principle from the few cases that have come under my own observation; yet as far as so small a number go, they add the influence of their results to this latter view.

The hazards of epilepsy and insanity alone, to say nothing of the more pressing danger of inflammation, abscess, and compression, I think, would not only justify, but imperiously demands the removal of pressure. While a patient is remaining quiet, in bed, on low diet, and under the effects of blood-letting and purgatives, he might with impunity bear an amount of pressure that could not be tolerated at all when he should be permitted to resume a full diet, and engage in an active life. There is an abundance of testimony that the most deplorable results may declare themselves, long years after the occurrence of the injury and the semi-recovery. I am acquainted with a young lady, a raving maniac, who sustained an injury of the os frontis in infancy; at puberty she manifested symptoms of insanity at her catamenial period, with intense pain in the forehead and excessive conjunctival redness. I advised trephining in her case, but it was not submitted to, and she has for some years been a confirmed lunatic I know another case, that of a young man, who, ten years after receiving an injury of the skull at the upper right lateral portion of the os frontis, became subject to epilepsy about once a month, with insanity for two or three days preceding and succeeding the epileptic seizure. In this case also, I advised the use of the trephine; but it was not agreed to, and the case remains as it was, except that it is growing gradually worse. These cases of course, I only conjecture to have been caused by spicula of bone; as the operation has not been performed, the correctness of the diagnosis has not been verified. But surgical books are full of such cases, in which this condition of things has been established by operations or by autopsies.

If the doctrine inculcated, to wait for constitutional symptoms to declare themselves, before resorting to an operation be erroneous and unsafe, as I believe it is, then great injury must result from it. An experienced surgeon may be untrammeled by authorities; he may, and will treat each individual case as he thinks proper, regardless of what others may have written; but it is otherwise with many into whose hands cases of this kind are committed for treatment. I know of no village that does not contain physicians entirely competent to perform this operation with the utmost safety; but the fear of operating unnecessarily may often induce them to postpone it to so late a period, that an operation can do no good. It is most important, that these should have their minds imbued with the proper principles for their guidance in such circumstances—important as it regards the safety of their patients; and important as it regards their own good name.

I would then, in conclusion, propose the following rule: That in every clearly ascertained fracture of the cranium, *with depression*, the bone should be elevated or removed, whether there be a scalp-wound or not; and I would add this suggestion, that where there is no wound in the integuments and this is to be made by incision, it is better to make it in a V shape; because this will give a sound flap for the covering of the exposed brain, which is to be preferred to that coming together by four points, over the centre of the wound through the skull, as is the case with the flaps made by the crucial incision.

Louisville, Feb. 23d, 1852.

